# Supplementary material for: Fungal Secondary Metabolite Exophillic Acid Selectively Inhibits the Entry of Hepatitis B and D Viruses
Source: Viruses. 2022 Apr 6;14(4):764. doi: 10.3390/v14040764 (PMC9026752; doi:10.3390/v14040764)
Supplement: Supplementary file 1 [file viruses-14-00764-s001.zip › viruses-1645548-supplementary.pdf]

Supporting information

Figure S1. Original images for immunoblots

Original images for immunoblots for Figure 6A

Text S1. General information of compounds preparation

Text S2. Fermentation and isolation of Exophillic acid and its analog

Figure S2. Preparation of Exophillic acid derivatives

(A) Preparation of Exophillic acid-propargylamide

(B) Preparation of Exophillic acid-Triazole-PEG_3-Biotin

Figure S3. NMR spectra of compounds

(A) ^{1}H NMR spectrum of Exophillic acid in pyridine-d_5

(B) ^{13}C NMR spectrum of Exophillic acid in pyridine-d_5

(C) ^{1}H NMR spectrum of TPI-1 in CD_3OD

(D) ^{13}C NMR spectrum of TPI-1 in CD_3OD (between 0 and 200 ppm)

(E) ^{13}C NMR spectrum of TPI-1 in CD_3OD (Expanded between 60 and 180 ppm)

(F) ^{13}C NMR spectrum of TPI-1 in CD_3OD (Expanded between 11 and 50 ppm)

(G) ^{1}H NMR spectrum of TPI-2 in CD_3OD

(H) ^{13}C NMR spectrum of TPI-2 in CD_3OD (between 0 and 200 ppm)

(I) ^{13}C NMR spectrum of TPI-2 in CD_3OD (Expanded between 60 and 175 ppm)

(J) ^{13}C NMR spectrum of TPI-2 in CD_3OD (Expanded between 12 and 52 ppm)

(K) ^{1}H NMR spectrum of Exophillic acid-propargylamide in pyridine-d_5

(L) ^{13}C NMR spectrum of Exophillic acid-propargylamide in pyridine-d_5

(M) ^{1}H NMR spectrum of Exophillic acid-Triazole-PEG_3-Biotin in pyridine-d_5

(N) ^{13}C NMR spectrum of Exophillic acid-Triazole-PEG_3-Biotin in pyridine-d_5

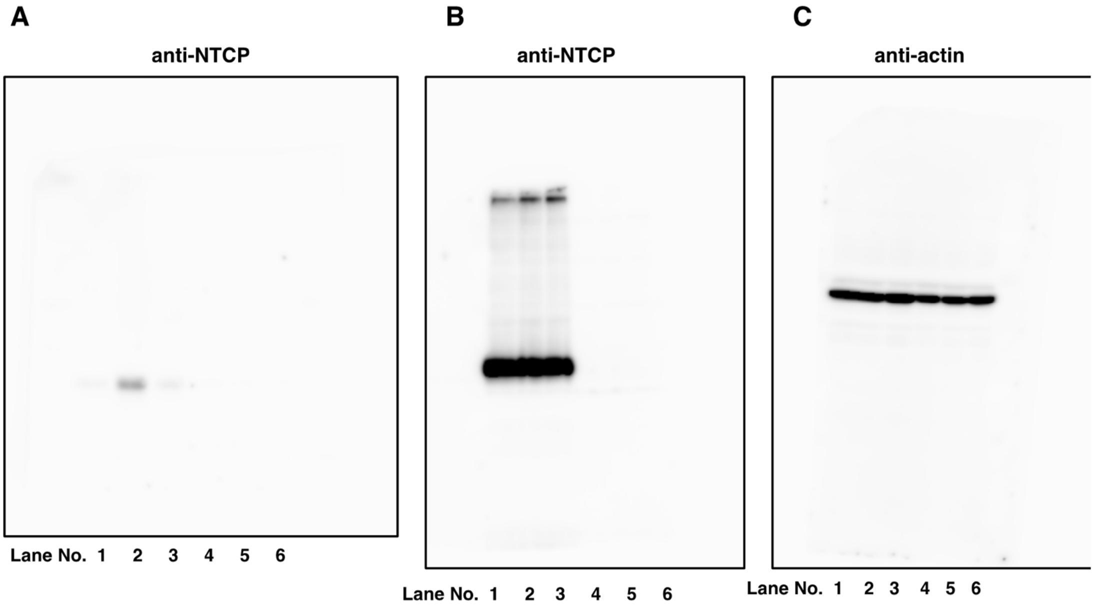

Immunoblot original images for Figure. 6A

Figure S1. Original images for immunoblots. The raw data of immunoblots shown in Figure 6A. HepG2-hNTCP-C4 (lanes 1-3) or HepG2 (lanes 4-6) cells incubated with biotinylated exophillic acid (lanes 2, 3, 5, and 6) or left untreated (lanes 1 and 4) were used for the pull down assay. In lane 3 and 6, pull down assay was performed in the presence of excess amount of non-biotinylated exophillic acid. (**A**), (**B**), and (**C**) correspond to the raw data of upper, middle and lower panels shown in Figure 6A.

Text S1. General information of compound preparation.

Unless otherwise noted, the reagents and solvents used here were commercially available and used without further purification. All solvents such as DMF and DMSO were purchased from Kanto Chemical. For thin-layer chromatography (TLC) analysis, precoated silica gel plates with a fluorescent indicator (60 F_{254}, Merck) were used. Thin-layer chromatography (TLC) was carried out with E. Merck silica gel plates (60F-254, 0.25 mm). Flash chromatography was performed with spherical neutral, 0.040–0.050-mm 60 N silica gels (Kanto Chemical). Optical rotations were measured using the JASCO P-2200 polarimeter. Infrared (IR) spectra were recorded using the JASCO FT-/IR-4600 spectrometer. NMR spectra were measured on a JEOL JNM-ECA-500 spectrometer with ^{1}H NMR at 500 MHz and ^{13}C NMR at 125 MHz or a Varian XL-400 spectrometer (Agilent Technologies, CA, USA) with ^{1}H NMR at 400 MHz and ^{13}C NMR at 100 MHz, respectively. Chemical shifts were reported from the internal solvent peaks for CD_3OD (^{1}H, δ = 3.31 ppm; ^{13}C, δ = 49.00 ppm), pyridine-d_5; (^{1}H, δ = 8.74 ppm; ^{13}C, δ = 150.35). ^{1}H NMR data were reported as follows: chemical shift (integration, multiplicity (s = singlet, d = doublet, t = triplet, m = multiplet, br = broad), coupling constants [Hz]). The high-resolution mass spectra (HRMS) were obtained using a JEOL JMS-700 MStation and JEOL JMS-T100LP. The HPLC system used was a pump SSC-3465 (Senshu Scientific), UV detector SSC-5410 (Senshu Scientific), preparative column PEGASIL ODS SP100 (20 i.d. × 250 mm (Senshu Scientific)), and Capcell Pak C_{18} (20 i.d. × 250 mm (Osaka Soda)).

DCC: *N,N'*-dicyclohexylcarbodiimide

DMAP: dimethyl-4-aminopyridine

HOBt: 1-Hydroxybenzotriazole

DMF: *N,N*-dimethylformamide

$[Cu(CH_3CN)_4]PF_6$: Tetrakis(acetonitrile)copper(I) Hexafluorophosphat

PEG: Polyethylene Glycol

DMSO: Dimethyl Sulfoxide

Text S2. Fermentation and isolation of Exophillic acid and its analog.

Exophillic acid: The fungal strain *Exophila* sp. FKI-7082 was grown and maintained on an agar slant consisting of 0.1% glycerol, 0.08% KH_2PO_4, 0.02% K_2HPO_4, 0.02% $MgSO_4{\cdot}7H_2O$, 0.02% KCl, 0.2% $NaNO_3$, 0.02% yeast extract and 1.5% agar (adjusted to pH 6.0 before sterilization). A loopful of spores of the strain was inoculated into 100 mL of the seed medium consisting of 2.0% glucose, 0.5% Hipolypepton (Nihon Pharmaceutical Co., Japan), 0.2% yeast extract, 0.2% KH_2PO_4, 0.05% $MgSO_4{\cdot}7H_2O$, and 0.1% agar (adjusted to pH 6.0 before sterilization) in each 500 mL-Erlenmeyer flask. The flask was incubated on a rotary shaker (210 rpm) at 27°C for 3 days. One milliliter of the seed culture was inoculated into each of ten 500 mL-Erlenmeyer flasks containing 100 mL of production medium (3.0% sucrose, 3.0% soluble starch, 1.0% malt extract, 0.3% Ebios (Asachi Group Foods Ltd.), 0.5% KH_2PO_4, 0.05% $MgSO_4{\cdot}7H_2O$, and 0.5% agar). Production cultivation was performed in a rotary shaker (210 rpm) at 27°C for 3 days, followed by static fermentation at 25°C for 10 days.

The culture broth (1 L) was extracted with acetone (1 L). After the centrifuged (3,800 rpm, 10 min), and the removal of acetone *in vacuo*. The obtained aqueous solution (0.5 L) was extracted by ethyl acetate (1 L, 3 times) and concentrated to dryness. Ethyl acetate extract (496 mg) was dissolved with 95% MeOH aq. (3 mL) and washed with *n*-hexane (3 mL, 2 times). The 95% MeOH aq. Layer was applied to Sep-pak C_{18} light (Waters Co. Ltd.), and eluted with MeOH (1.5 mL). 95% MeOH aq. And MeOH fractions (4.5 mL) underwent HPLC using Capcell Pak C_{18} (20 i.d. x 250 mm, Osaka Soda Co. Ltd, Osaka, Japan) with an isocratic solvent system of MeOH-Trifluoroacetic acid (100:0.05) at a flow rate of 7.0 mL/min detected by UV 210 nm. The fraction with retention time of 11-12 min were collected, added water, evaporated *in vacuo* to remove MeOH, and freeze-dried to afford Exophilic acid (125.1 mg). Exophillic acid was identified by ^{1}H and ^{13}C NMR spectra measured in pyridine-d_5 (Figs. S4-1 and -2) and a $[M+H]^+$ ion with *m/z* 705 in ESI-MS analysis.

TPI-1 and TPI-2: The fungal strain *Exophila* sp. FKI-6150 was cultured under the same conditions as FKI-7082 except for the production medium. One milliliter of the seed cultures of FKI-6150 was inoculated into each of nineteen 500 mL-Erlenmeyer flasks containing 100 mL of production medium (2.0% sucrose, 1.0% glucose, 0.5% corn steep powder, 0.5% meat extract, 0.1% KH_2PO_4, 0.3% $CaCO_3$, and 0.1% agar). The cultured broth (1.9 L) was extracted with EtOH (1.9 L) and removed EtOH *in vacuo*. The obtained aqueous solution was extracted by ethyl acetate and concentrated to dryness. Ethyl acetate extract (5.7 g) was applied to a silica gel column chromatography and eluted stepwise with $CHCl_3$/MeOH = 100/0, 100/10, 100/50, 50/50 and 0/100 (each 500 mL). TPI-1 and -2 were eluted with 100/10, 100/50 and 50/50 fractions, and concentrated *in vacuo* to dryness.100/10 fraction was purified with preparative TLC ($CHCl_3$/2-propanol/H_2O/acetic acid =100/40/5/1) to afford TPI-1 (30 mg) and -2 (40 mg), respectively. TPI-1 and -2 were identified by ^{1}H and ^{13}C NMR spectra measured in CD_3OD (Figs. S4-3, -4, -5, and -6) and a $[M+H]^+$ ion with *m/z* 649 in ESI-MS analysis.

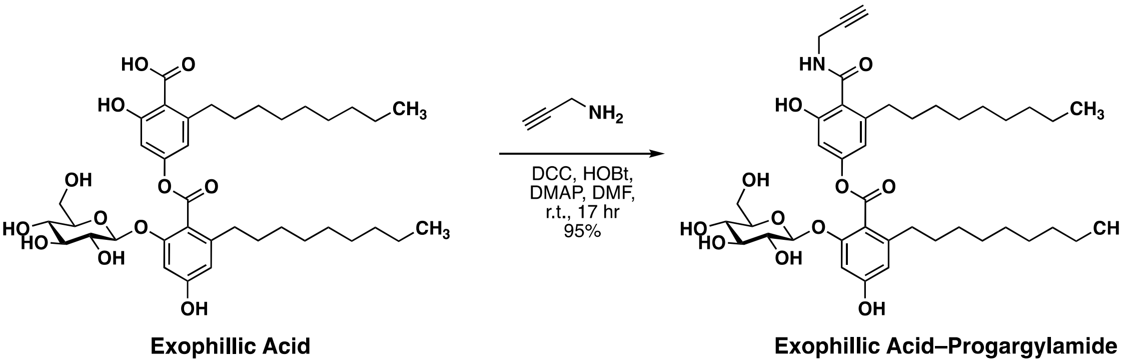

(**A**) Preparation of Exophillic acid-Propargylamide

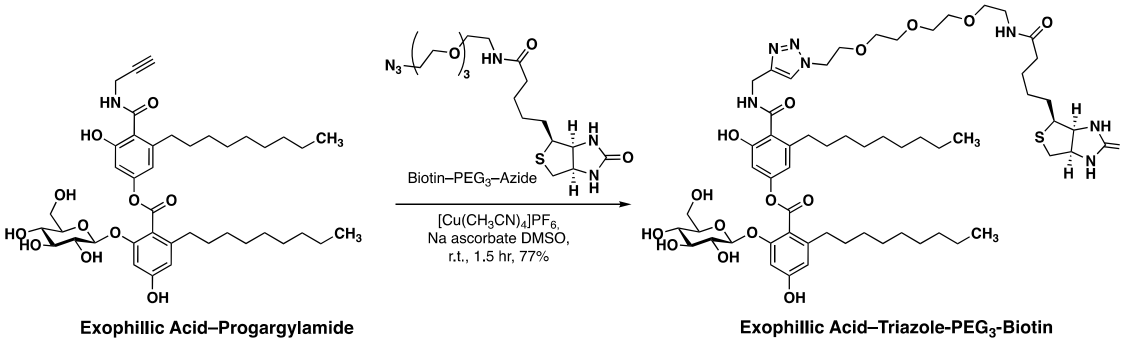

(**B**) Preparation of Exophillic acid–Triazole–PEG_3–Biotin

Figure S2. Preparation of Exophilic acid derivatives. (**A**) Exophillic acid-Propargylamide. (**B**) Exophillic acid-Triazole-PEG_3-Biotin.

To a solution of **Exophillic acid** (naturally occurring compound) (10.0 mg, 0.0142 mmol) in DMF (0.71 mL) at room temperature were added HOBt (7.7 mg, 0.0567 mmol), DMAP (3.5 mg, 0.0284 mmol), propargylamine (4.0 μL, 0.0624 mmol) and DCC (11.7 mg, 0.0567 mmol). After stirring for 17 hours, the reaction was quenched by the addition of NH_4Cl aq. (5 mL), and the resulting mixture was extracted with $CHCl_3$ (10 mL x 3). The combined organic layers were dried over Na_2SO_4, filtered and concentrated. The crude product was purified by flash column chromatography on silica gel ($CHCl_3$/MeOH = 2/1 v/v) to provide **Exophillic acid–propargylamide** (10.0 mg, 95%) as a colorless amorphous solid. Physico-chemical properties: IR (diamond prism) ν cm^{-1}: 3283, 2925, 2854, 2361, 1724, 1589, 1530, 1462, 1309, 1246, 1168, 1147, 1130, 1053, 891, 853. HRMS (ESI) *m/z* 764.4022 $[M+Na]^+$, calcd. 764.3986 for $C_{41}H_{59}NO_{11}Na^+$, $[\alpha]^{22}_D$; -13.8 (*c* = 0.1, MeOH). ^{1}H NMR (500 MHz, pyridine-d_5, Fig. S4-7) δ 10.03 (d, *J* = 5.2 Hz, 1H), 7.36 (d, *J* = 2.0 Hz, 1H), 7.33 (d, *J* = 2.2 Hz, 1H), 7.15 (d, *J* = 2.1 Hz, 1H), 6.92 (d, *J* = 2.0 Hz, 1H), 5.62 (d, *J* = 7.3 Hz, 1H), 4.72 (s, 1H), 4.69 – 4.64 (m, 2H), 4.34 (dd, *J* = 3.6, 2.3 Hz, 2H), 4.31 – 4.24 (m, 3H), 4.06 (d, *J* = 10.8 Hz, 1H), 3.92 (m, 1H), 3.22 (t, *J* = 2.5 Hz, 1H), 3.00 – 2.91 (m, 3H), 2.07 (d, *J* = 12.1 Hz, 2H), 1.83 (dt, *J* = 9.8, 7.7 Hz, 2H), 1.58 (d, *J* = 10.8 Hz, 2H), 1.38 (m, 2H), 1.32 (m, 4H), 1.26 – 1.14 (m, 16H), 0.86 (t, *J* = 8.7 Hz, 3H) 0.86 (t, *J* = 8.7 Hz, 3H). ^{13}C NMR (125 MHz, pyridine-d_5, Fig. S4-8) 168.8, 168.0, 162.2, 158.5, 157.3, 153.2, 144.4, 144.1, 125.4, 115.7, 114.3, 111.8, 108.8, 103.4, 102.3, 82.3, 79.3, 79.1, 75.3, 72.9, 71.3, 62.6, 52.8, 34.8, 34.4, 33.7, 33.7, 32.6, 32.4, 32.1, 30.5, 30.4, 30.4, 30.3, 30.3, 30.1, 29.9, 23.4, 23.4, 14.8, 14.7

To a solution of **Exophillic acid–propargylamide** (13.9 mg, 0.0187 mmol) in DMSO (1.9 mL) at room temperature were added biotin–PEG_3–azide (12.5 mg, 0.0281 mmol), $[Cu(CH_3CN)_4]PF_6$ (1.4 mg, 3.75 µmol) and sodium ascorbate (5.6 mg, 0.0281 mmol). After stirring for 1.5 hours, the reaction was diluted by the addition of NaCl aq. (8 mL), and the resulting mixture was extracted with $CHCl_3$ (15 mL x 3). The combined organic layers were dried over Na_2SO_4, filtered and concentrated. The crude product was dissolved in CH_3CN and the insoluble components were removed by filtration. The filtrate was concentrated and purified by HPLC (90% MeOH aq. + 0.05% TFA) to provide **Exophillic acid-Triazole-PEG$_3$-Biotin** (17.1 mg, 77%) as a colorless amorphous solid. Physico-chemical properties: IR (diamond prism) ν cm^{-1}: 3281, 2924, 2855, 1668, 1588, 1548, 1462, 1428, 1310, 1245, 1164, 1127, 1050, 890. HRMS (ESI) *m/z* 1186.6299 $[M+H]^+$, calcd. 1186.6321 for $C_{59}H_{92}N_7O_{16}S^+$, $[\alpha]^{23}_D$; -3.8 (*c* = 0.1, MeOH). ^1H NMR (500 MHz, 7yridine-*d*$_5$. Fig. S4-9) δ 10.02 (t, *J* = 5.7 Hz, 1H), 8.59 (s, 1H), 8.33 (s, 1H), 7.55 (s, 1H), 7.35 (d, *J* = 2.1 Hz, 1H), 7.30 (d, *J* = 2.1 Hz, 1H), 7.12 (d, *J* = 2.1 Hz, 1H), 6.91 (d, *J* = 2.1 Hz, 1H), 5.60 (d, *J* = 7.4 Hz, 1H), 5.17 (d, *J* = 5.6 Hz, 2H), 4.59 (t, *J* = 5.2 Hz, 2H), 4.53 (dd, *J* = 7.7, 4.7 Hz, 1H), 4.38 – 4.28 (m, 3H), 4.28 – 4.18 (m, 3H), 3.94 – 3.87 (m, 1H), 3.84 (t, *J* = 5.2 Hz, 2H), 3.67 (d, *J* = 2.8 Hz, 4H), 3.64 – 3.55 (m, 4H), 3.54 (s, 2H), 3.18 (ddd, *J* = 8.2, 6.3, 4.3 Hz, 1H), 3.02 – 2.82 (m, 5H), 2.36 (t, *J* = 7.3 Hz, 2H), 1.95 – 1.71 (m, 9H), 1.56 (dt, *J* = 15.5, 7.3 Hz, 2H), 1.44 – 1.28 (m, 6H), 1.20 (d, *J* = 8.4 Hz, 17H), 0.86 (t, *J* = 7.0 Hz, 3H), 0.86 (t, *J* = 7.0 Hz, 3H). ^{13}C NMR (125 MHz, pyridine-*d*$_5$, Fig. S4-10) δ 173.8, 169.2, 168.0, 165.1, 162.2, 158.5, 157.3, 153.2, 146.6, 144.5, 144.0, 125.6, 124.3, 115.7, 114.3, 111.8, 108.8, 103.4, 102.3, 79.4, 79.0, 75.4, 71.3, 71.2, 71.1, 71.0, 70.9, 70.2, 62.9, 62.6, 61.1, 56.8, 50.8, 41.5, 40.3, 37.0, 36.6, 36.6, 36.4, 34.8, 34.4, 32.6, 32.6, 32.6, 32.4, 32.1, 30.5, 30.5, 30.4, 30.4, 30.4, 30.1, 29.5, 29.4, 26.6, 23.4, 23.4, 14.8, 14.7.

Figure S3. NMR spectra of compounds.

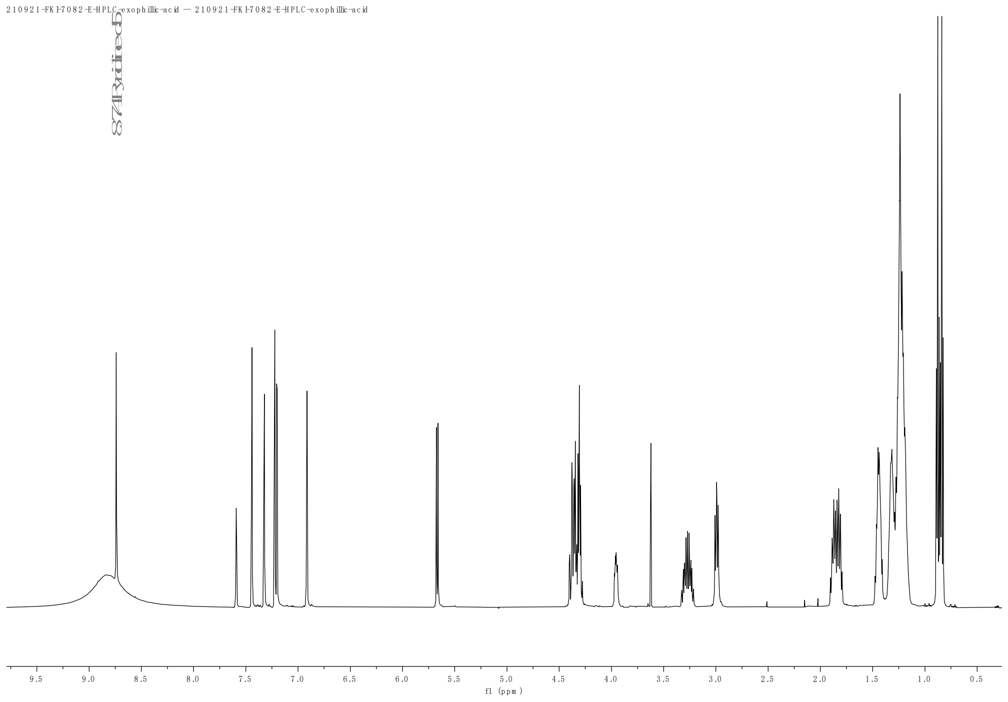

(**A**) ^{1}H NMR spectrum of Exophillic acid in pyridine-d_5

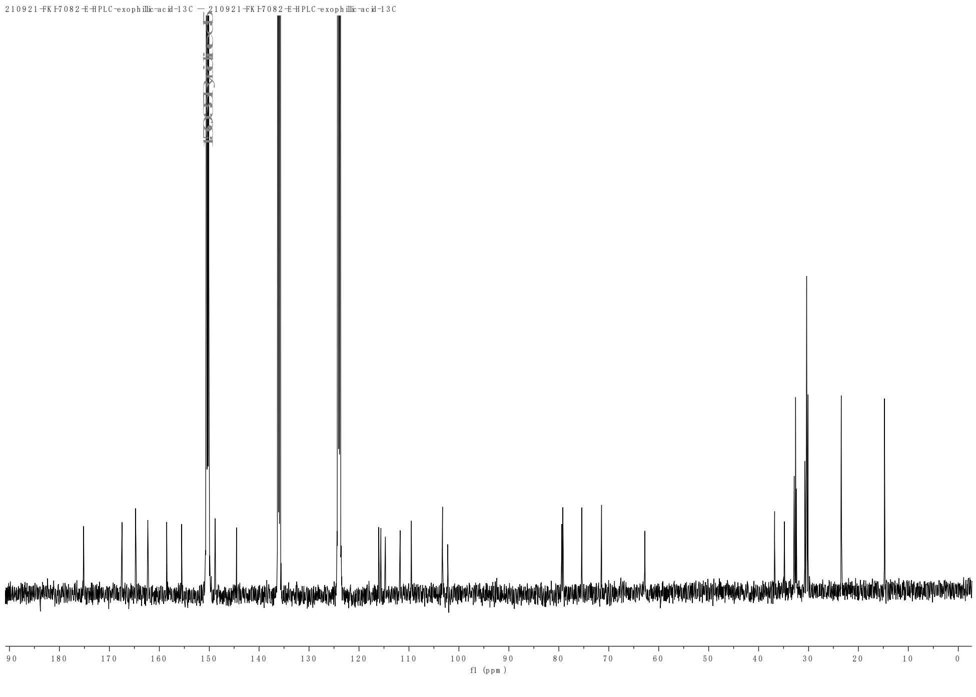

(**B**) ^{13}C NMR spectrum of Exophillic acid in pyridine-d_5

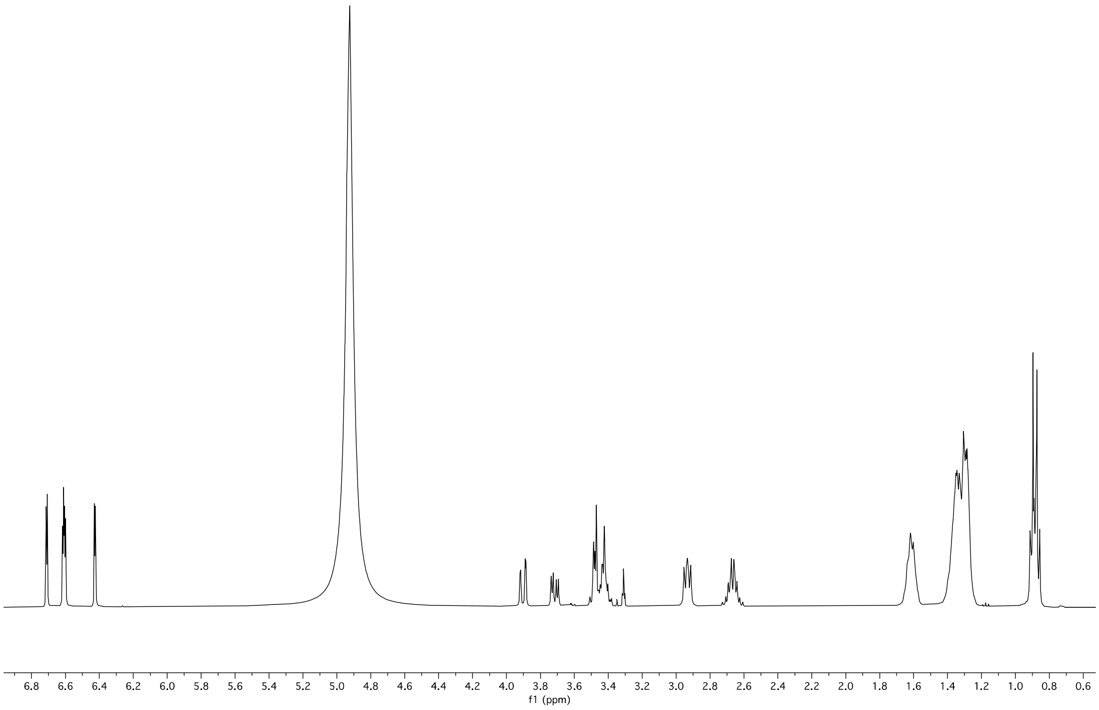

(**C**) ^{1}H NMR spectrum of TPI-1 in CD_3OD

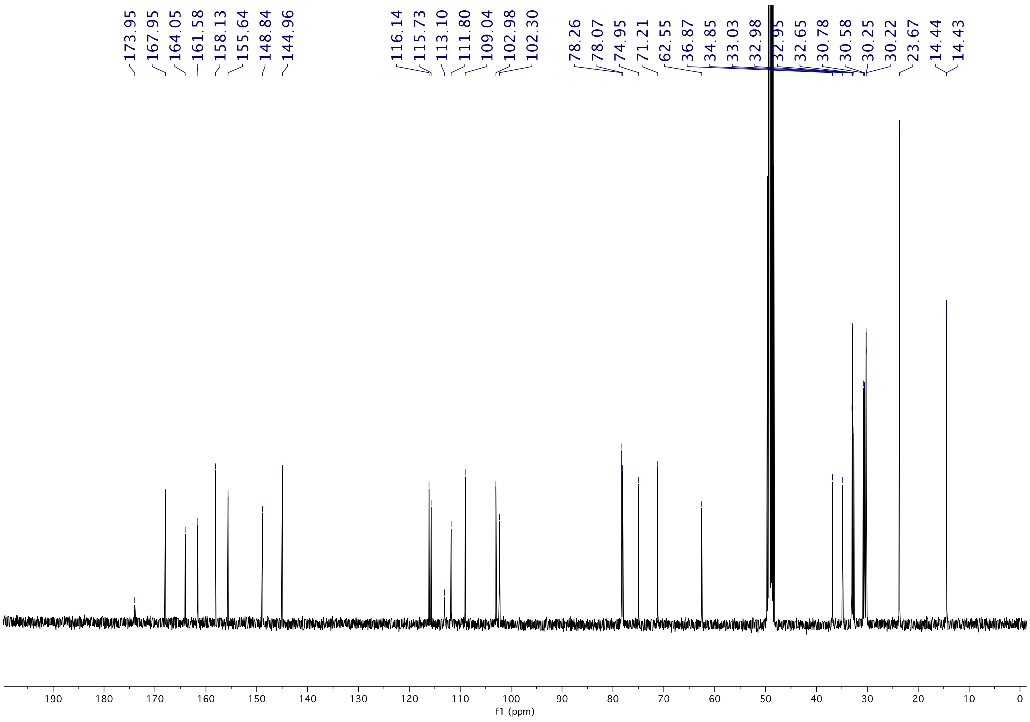

(**D**) ^{13}C NMR spectrum of TPI-1 in CD_3OD (between 0 and 200 ppm)

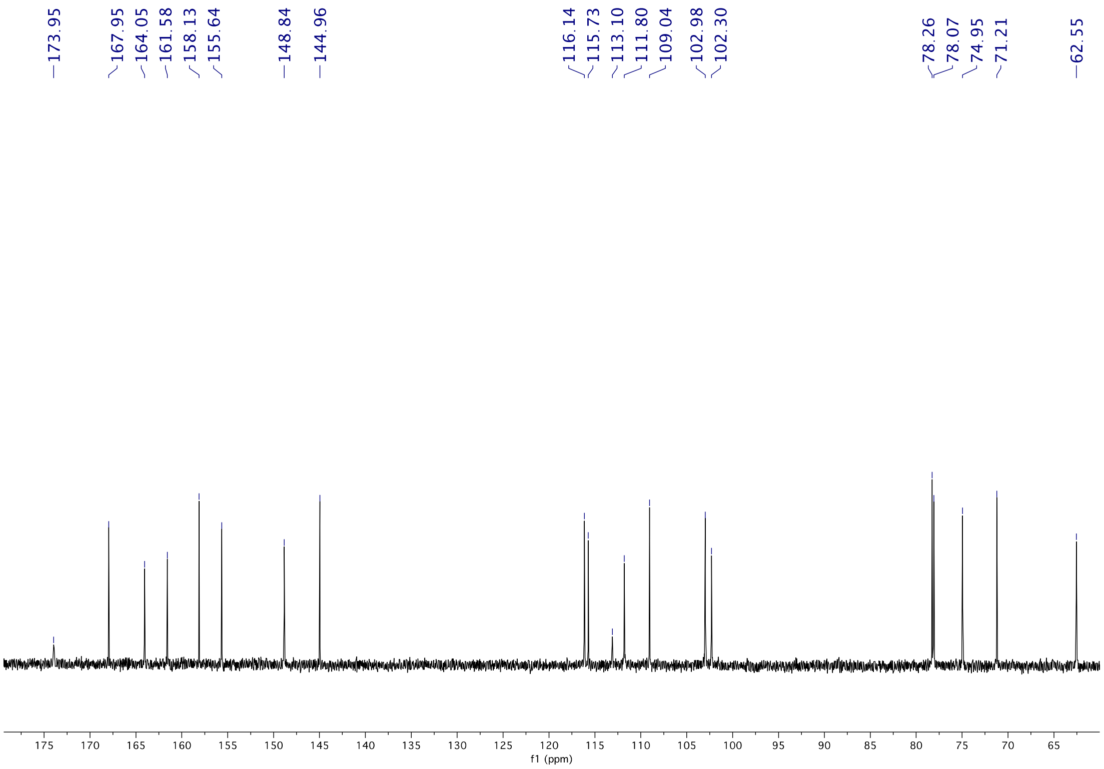

(**E**) ^{13}C NMR spectrum of TPI-1 in CD_3OD (Expanded between 60 and 180 ppm)

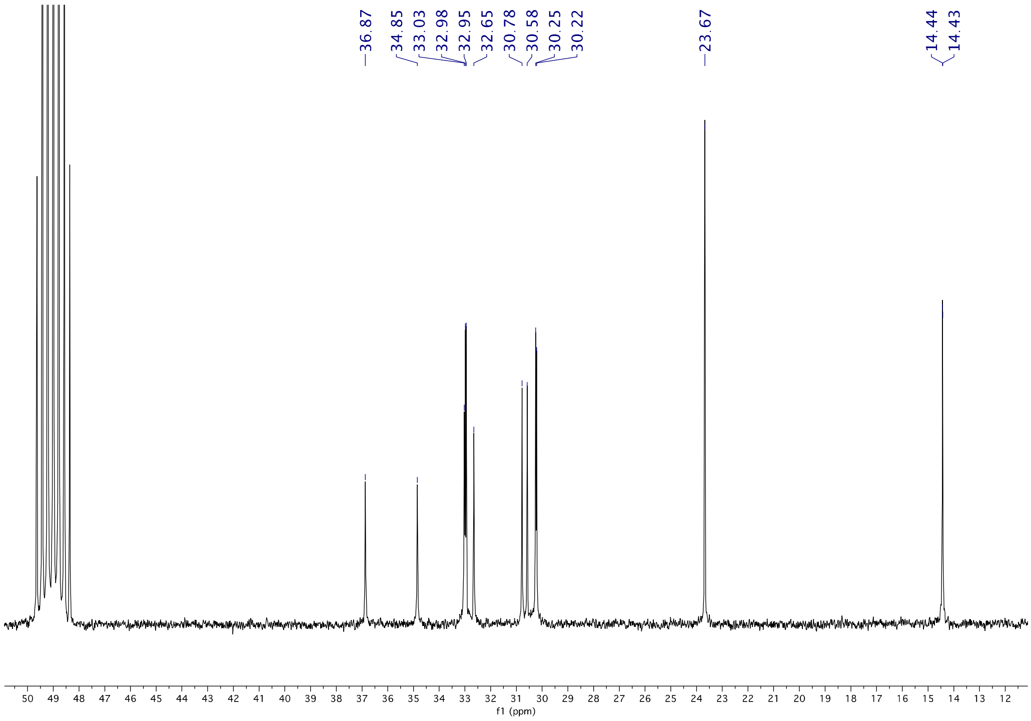

(**F**) ^{13}C NMR spectrum of TPI-1 in CD_3OD (Expanded between 11 and 50 ppm)

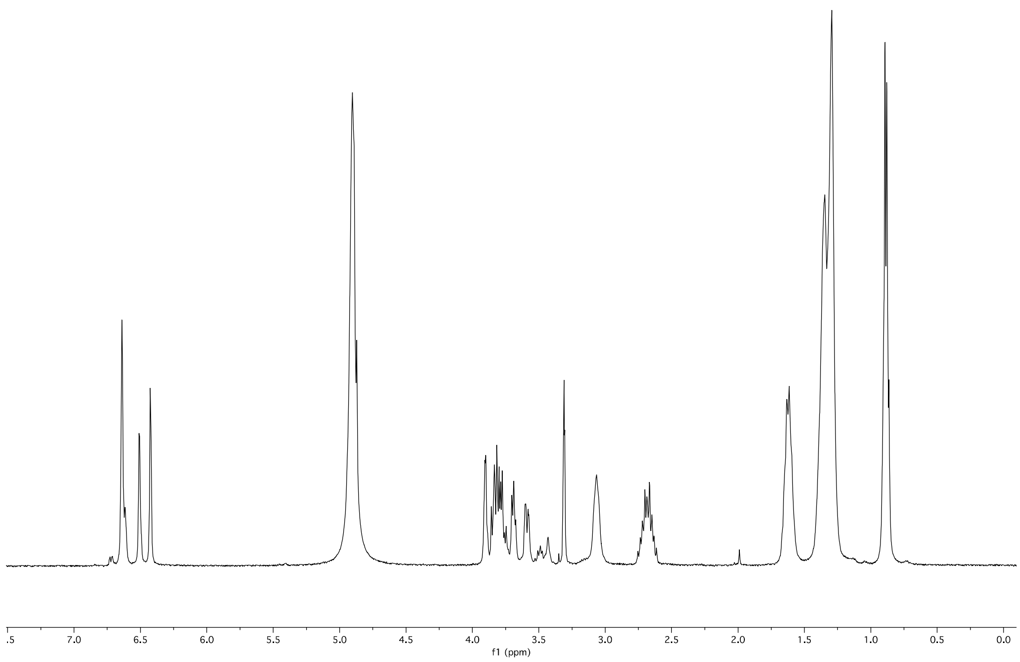

(**G**) ^{1}H NMR spectrum of TPI-2 in CD_3OD

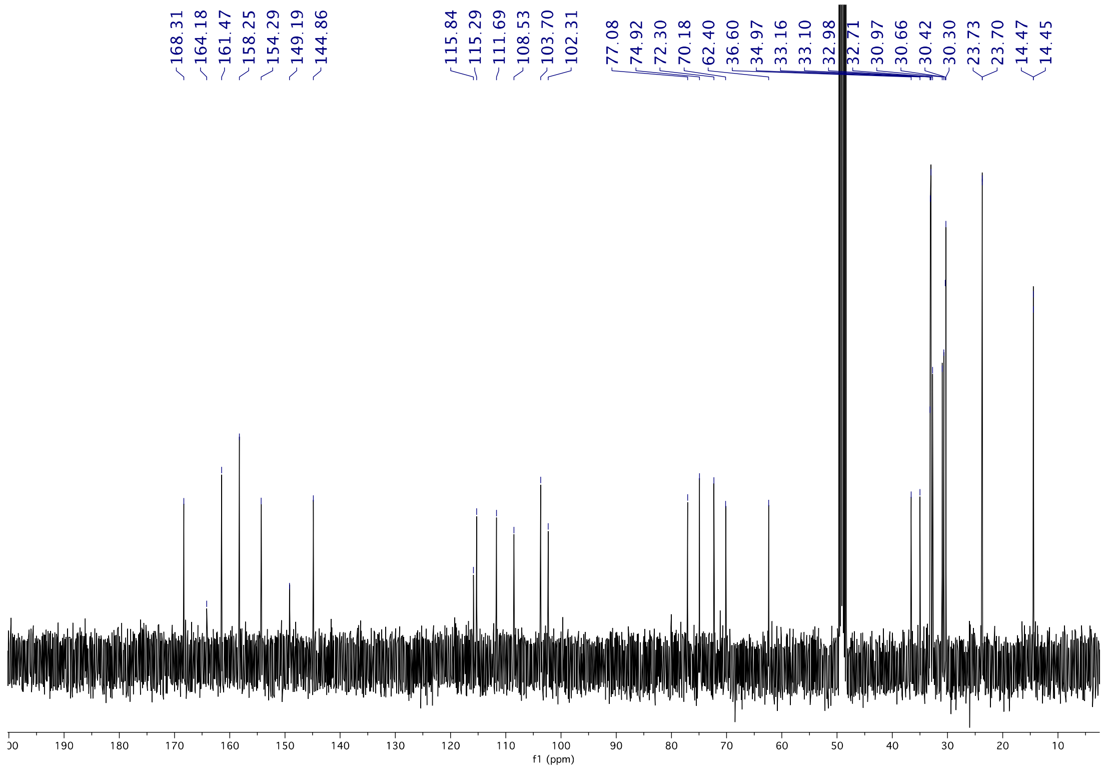

(**H**) ^{13}C NMR spectrum of TPI-2 in CD_3OD (between 0 and 200 ppm)

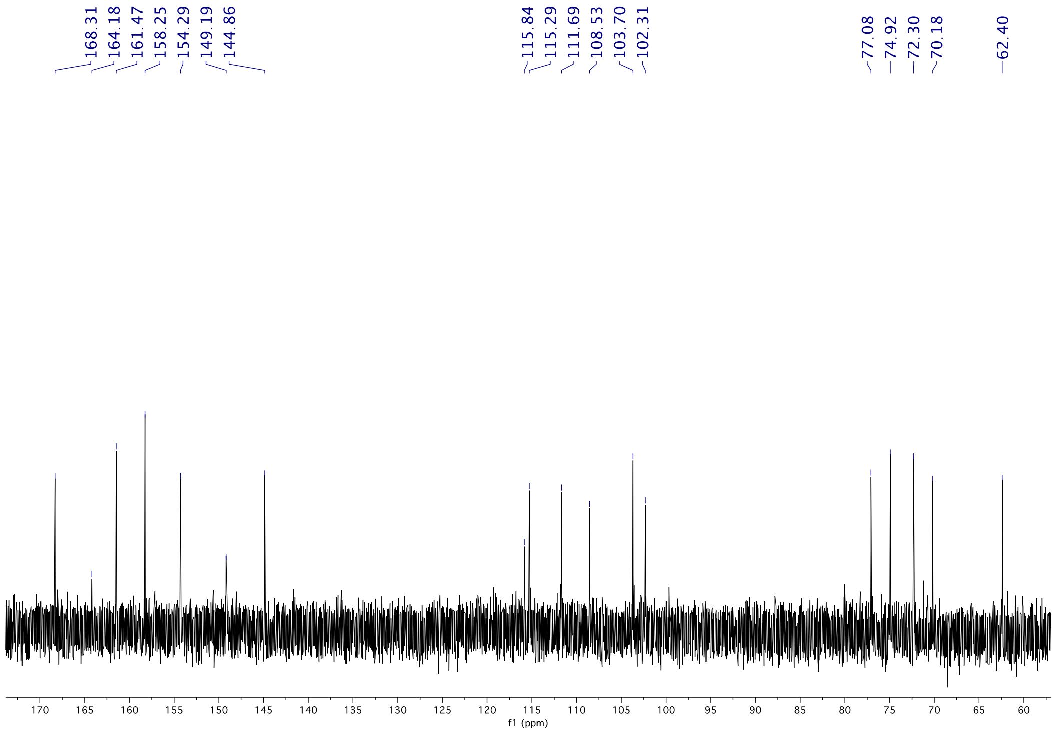

(**I**) ^{13}C NMR spectrum of TPI-2 in CD_3OD (Expanded between 60 and 175 ppm)

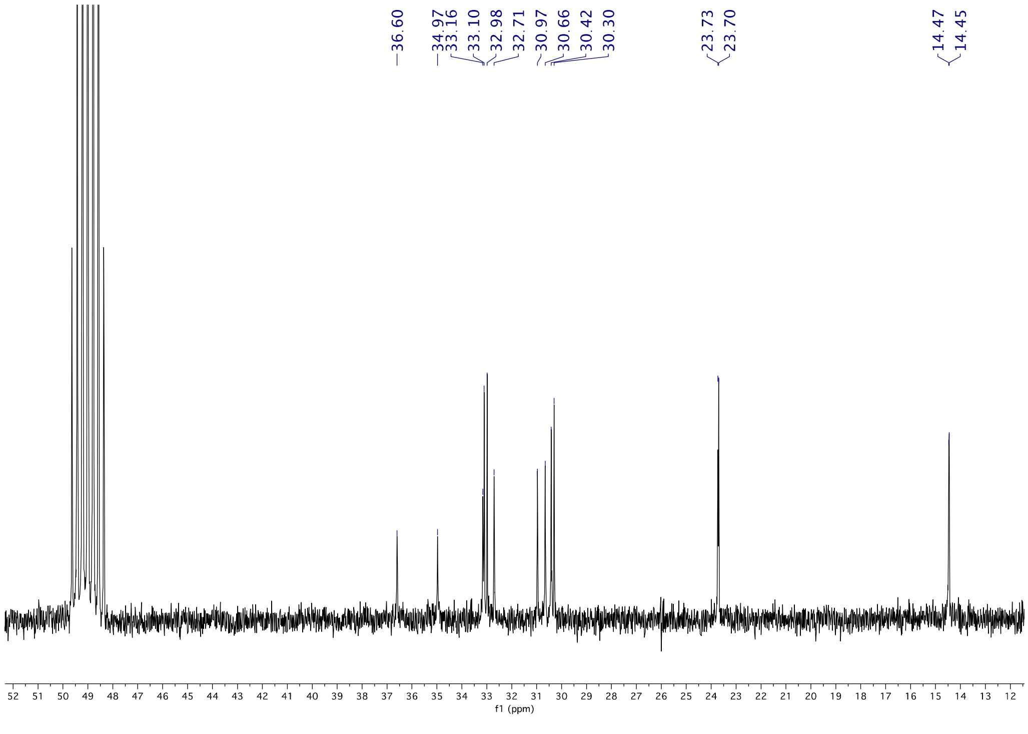

(**J**) ^{13}C NMR spectrum of TPI-2 in CD_3OD (Expanded between 12 and 52 ppm)

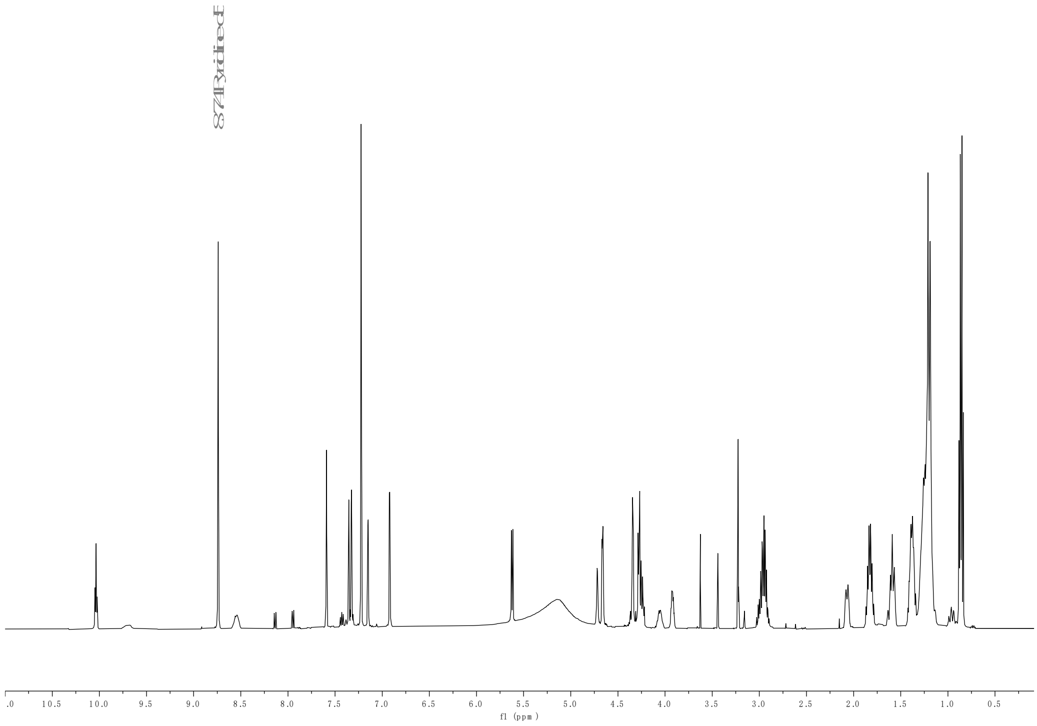

(**K**) ^{1}H NMR spectrum of Exophillic acid-propargylamide in pyridine-d_5

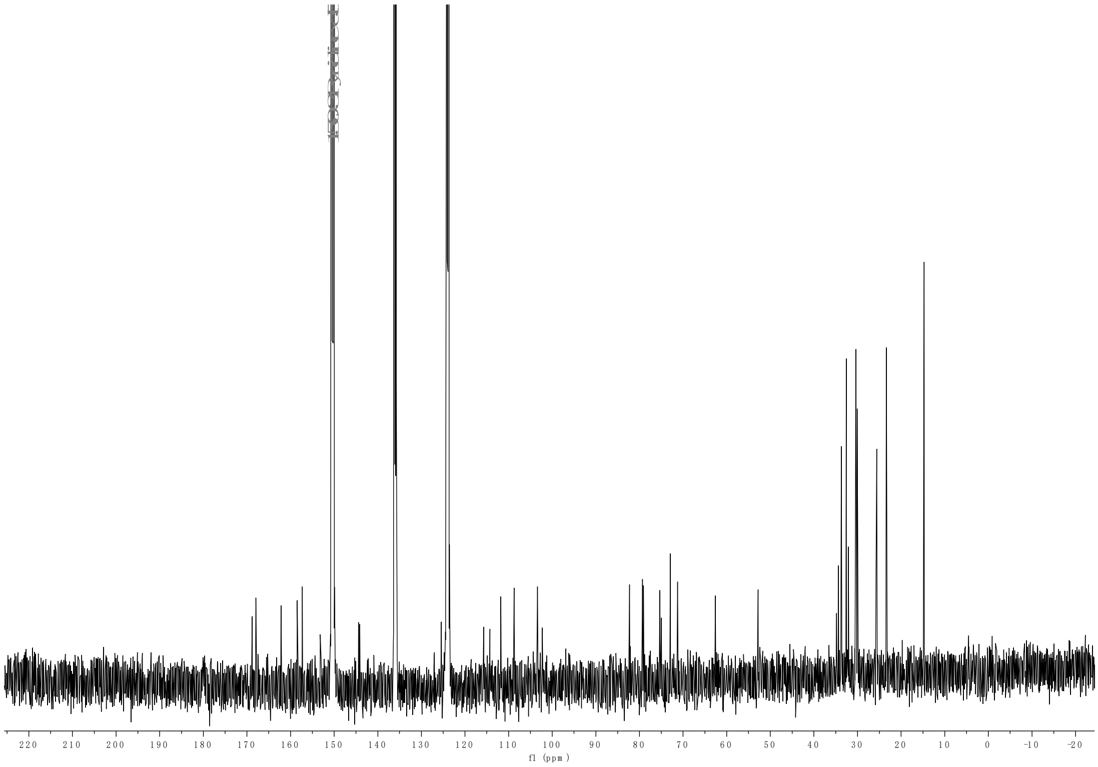

(**L**) ^{13}C NMR spectrum of Exophillic acid-propargylamide in pyridine-d_5

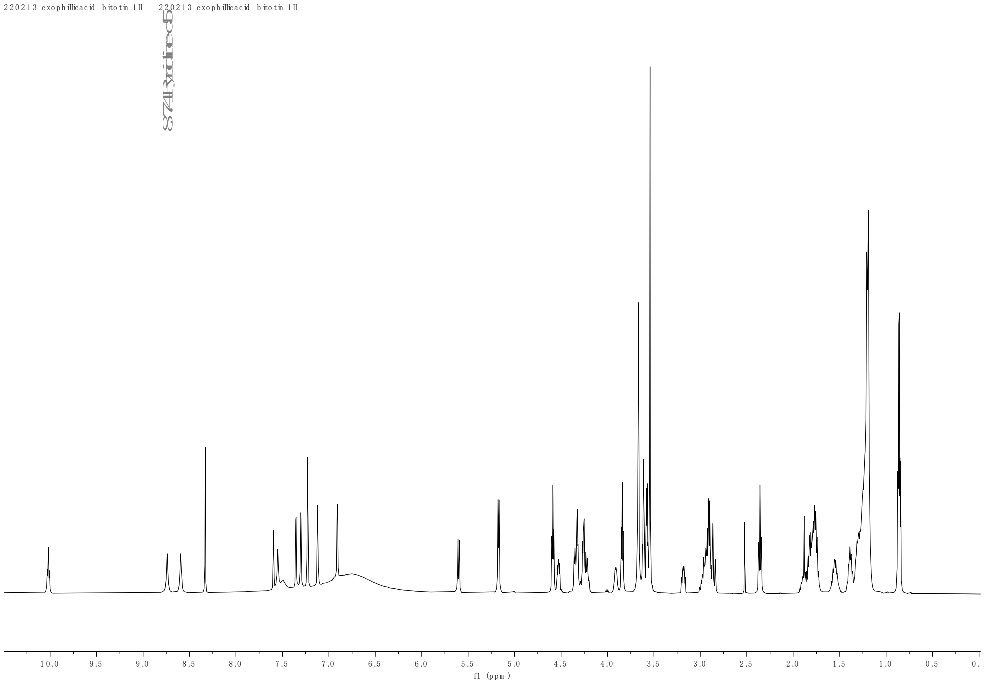

(**M**) ^{1}H NMR spectrum of Exophillic acid-Triazole-PEG$_3$-Biotin in pyridine-d_5

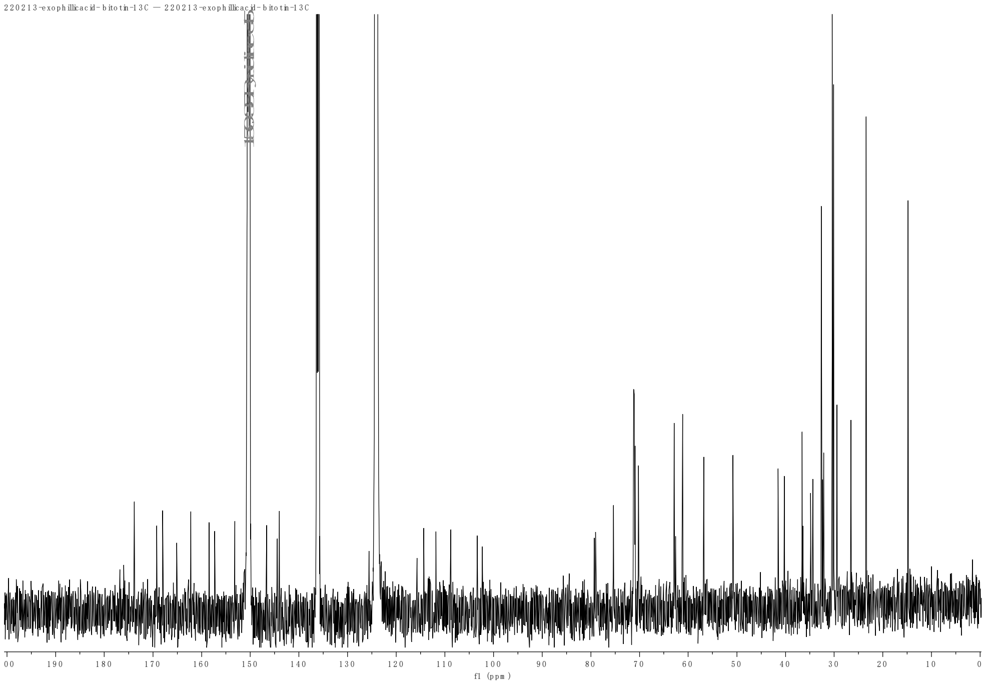

(**N**) ^{13}C NMR spectrum of Exophillic acid-Triazole-PEG$_3$-Biotin in pyridine-d_5
